# Error-free and error-prone DNA repair gene expression data through reprogramming and passage in human iPS cells

**DOI:** 10.1016/j.dib.2020.105228

**Published:** 2020-02-06

**Authors:** Yasuhide Yoshimura, Daisuke Okuzaki

**Affiliations:** a Division of Gene Therapy Science, Department of Genome Biology, Graduate School of Medicine, Osaka University, Japan; b Genome Information Research Center, Research Institute for Microbial Diseases, Osaka University, Japan

**Keywords:** DNA repair, PARP, RAD51, BLM, Homologous recombination, Mismatch repair, Reprogramming

## Abstract

We recently found that DNA repair-related gene expression could be altered by reprogramming as well as the increased expression of genes that accurately convey genomic information, such as homologous recombination (HR) and mismatch repair (MMR), and the decreased expression of error-prone translesion synthesis (TLS) polymerase. Here, we confirmed this change in expression in another cell-line and found that such alteration was maintained by overlapping passages as well as OCT3/4 and NANOG. Our findings suggest that changes in the expression of DNA repair-related genes associated with reprogramming and their maintenance can be novel indicators of the quality control of the cells exhibiting pluripotency.

**Table:** Specifications Table

Subject	*Biology*
Specific subject area	*NGS, Transcriptomics, Stem cell biology*
Type of data	*Table, figure*
How data were acquired	*Illumina sequencing (Illumina-HiSeq system)*
Data format	*Filtered and analyzed*
Parameters for data collection	*hiPSC lines were grown in serum-free human ESC medium. Total RNA from progenitor fibroblast and hiPS cells were extracted using commercial kits.*
Description of data collection	*RNA from progenitor cells and iPS cells subjected to RNA-Sequencing and transcriptome profiling. An Illumina Casava ver.1.8.2 software was used for the base calling. The FPKM values were calculated from the respective sequence data, and the analyses were performed using iDEP85* (http://bioinformatics.sdstate.edu/idep/).
Data source location	*Osaka, Japan*
Data accessibility	*NCBI accession number:*GSE134441 (https://www.ncbi.nlm.nih.gov/geo/query/acc.cgi?acc=GSE134441).
Related research article	*Yoshimura Y, Yamanishi A, Kamitani T, Kim J-S, Takeda J (2019) Generation of targeted homozygosity in the genome of human induced pluripotent stem cells. PLoS ONE 14(12):* e0225740.https://doi.org/10.1371/journal. pone.0225740

**Table:** 

**Value of the Data** • This work gives a deeper understanding of the basic characteristics of DNA repair-related genes in pluripotent cells with reprogramming and overlapping passage. • The data in this article shows that changes in the expression of hiPSC passage group (p31, p32) and hiPSC passage group (p50, p51, p53) were clearly shown by PCA. • The difference between hiPSC passage group (p31, p32) and hiPSC passage group (p50, p51, p53) was indicated at cell differentiation.

## 1 Data description

The mean RNA expression values of fibroblast and hiPSC (p31, p32) were calculated for DNA repair- and replication-related genes, as noted in our previous analysis [1]. As a result, a stable and approximately three-fold elevated expression through reprogramming was observed in all the hiPS cell (hiPSC)lines, compared with the progenitor cells for *RAD51* and *BLM* in HR, *MSH2* and *MSH6* in MMR and *PARP1* and *PARP2* in base excision repair (BER) which is a part of error-free repairs. *RAD50*, *NBN* and *MRE11* were involved in both the HR and the non-homologous end-joining (NHEJ). *MRE11* showed a slight elevation of expression, but there was no increase in expression of *RAD51* or *BLM*, similar to our previous findings. *RAD50* and *NBN* showed a minimal decrease in expression, consistent with the previous data [1] (Table 1).

**Table 1:** Comparison of RNA expression levels of parental fibroblast and hiPSC passage groups of (p31, p32) and (p50, p51, p53). The average value is shown. Statistical analysis was performed between fibroblast and hiPSC passage group of (p31, p32), and between hiPSC passage group of (p31, p32) and hiPSC passage group of (p50, p51, p53) using a Student's t test, analyzed by a Caleida Graph. Comparison between fibroblast and hiPSC passage group of (p31, p32) ** <0.01, *<0.05. Comparison between hiPSC passage group of (p31, p32) and hiPSC passage group of (p50, p51, p53) ◎◎ <0.01, ◎<0.05. Data are expressed as the mean ± SEM.

			PFKM value		
Symbol	Accession No.	Activity	fibroblast	iPSC P31-32	pSO-53
PARP1	M32721	BER	36.20	159.82**	180.02
PARP2	NM_005484	BER	16 83	22.67	27.94
RAD51	NM_002875	HR	8.92	22.02**	22.07
BLM	NM_000057	HR	2.41	6.84**	9.95
MSH2	NM_000251.2	MMR	14.63	45.69**	48.64
MSH6	NM_000179.2	MMR	21.58	53.08**	59.17
RAD50	NM_005732	HR, NHEJ	14.01	12.36	11.71
MRE11	NM_005590	HR, NHEJ	5.95	8.79	10.04
NBN	NM_002485	HR, NHEJ	22.30	9.21	14.53^∞^
XRCC4	NM_003401.5	NHEJ	11.12	7.33	6.63
XRCC5	NM_021141.4	NHEJ	124.88	172.58	208.53
XRCC6	NM_001469	NHEJ	242.30	253.63	332.51°
POLH	NM_001291970.2	TLS	5.73	4.34*	4.13
REV3L	NM_001286432.1	TLS	13.11	3.12*	330
POU5F1	NM_001173531	pluripotency	0.34	467.76**	397.08
NANOG	AB093576	pluripotency	0.00	58.07**	48.08
GAPDH	NM_002046	Housekeeping	3691.37	2065.05	2194.58

Although expression was slightly upregulated in *XRCC5* and *XRCC6*, that in *XRCC4* was downregulated; all their corresponding genes were involved in the NHEJ of the error-prone repair. In addition, *REV3L* and *POLH* of the polymerase representatives, thought to perform ambiguous post-replicative repairs, showed reduced expression. All these alterations in expression were the same as those shown in a series of DNA repair-related genes using microarray, with a completely separate fibroblast and third molar cell [1].

**Fig. 1:**
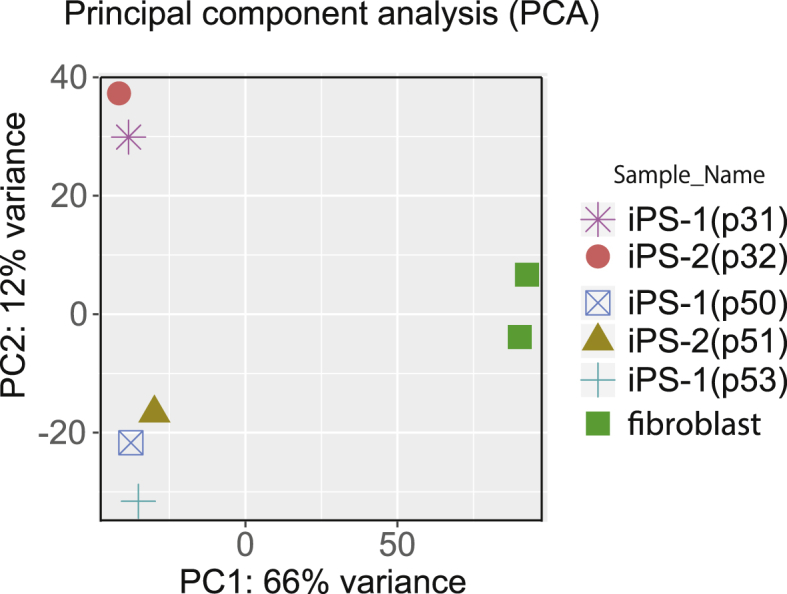
Principal component analysis of parental fibroblast and hiPSC passage groups of (p31, p32) and (p50, p51, p53).

The principal component analysis (PCA) showed that progenitor fibroblast and hiPSC were greatly divided by PC1 and PC2 and that the two passage groups of (p31, p32) and (p50, p51, p53) were divided in hiPSC (Fig. 1). Moreover, there were differences in the expression of genes between these two groups. We performed gene ontology (GO) analysis of the 761 genes, incurring a fold change of (2≧, ≦2) and obtaining p-values of <0.05 (p31, p32 vs p50, p51, p53). The top five GO associated genes included those for regulation of cell differentiation, positive regulation of developmental process, epithelium development, regulation of multicellular organismal development and epithelial cell differentiation (Table 2).

**Table 2:** The top five GO terms of differences in the expression of genes between iPSC cells groups of (p31, p32) and (p50, p51, p53).

	ID	Name	pValue	Genes from Input
1	GO:00455	regulation of cell differentiation	1.23E-10	104
2	GO:00510	positive regulation of developmental process	1.57E-10	87
3	G O:00604	epithelium development	6.88E-10	84
4	GO:20000	regulation of multicellular organismal development	1.14E-08	106
5	GO:00308	epithelial cell differentiation	2.53E-08	50

The mean values of each of the two groups were calculated for the FPKM values of OCT3/4 and NANOG as indices of pluripotency. No difference was found between the two groups, but our findings demonstrated that pluripotency was maintained even in the groups of (p50, p51, p53) compared with the groups of (p31, p32) (Table 1).

## 2 Experimental design, materials, and methods

### 2.1 Cell culture

hiPSC lines [2,3]were grown in hESC serum-free human ESC (hESC) medium consisting of DMEM/F-12 (Life Technologies) supplemented with 20% knockout serum replacement (Life Technologies), 2 mM l-glutamine, 1× nonessential amino acids (Life Technologies), 0.1 mM 2-mercaptoethanol, and 5 ng/mL basic fibroblast growth factor (Katayama Chemical Industries) on Synthemax II-SC-coated tissue culture dishes (Corning). The cells were passaged using Accutase (Sigma) and seeded with the Rho kinase inhibitor Y-27632 (10 μM; LC Laboratories).

### 2.2 RNA extraction and library preparation

Total RNA was extracted from cells with an RNeasy Plus Micro Kit (Qiagen). Library preparation was performed using a TruSeq stranded mRNA sample prep kit (Illumina, San Diego, CA) according to the manufacturer's instructions.

## 3 RNA sequence

Whole transcriptome sequencing was applied to the RNA samples through the Illumina HiSeq 2500 and 3000 platforms in a 75-base single-end mode. An Illumina Casava ver.1.8.2 software was used for the base calling. The sequenced reads were mapped to the human reference genome sequences (hg19) using TopHat ver. 2.0.13 in combination with Bowtie2 ver. 2.2.3 and SAMtools ver. 0.1.19. The number of fragments per kilobase of exon per million mapped fragments (FPKM) was calculated using Cufflinks ver. 2.2.1. The FPKM values were calculated from the respective sequence data, and the analyses were performed using iDEP85 (http://bioinformatics.sdstate.edu/idep/).
